# Human–Machine Multi-Turn Language Dialogue Interaction Based on Deep Learning

**DOI:** 10.3390/mi13030355

**Published:** 2022-02-23

**Authors:** Xianxin Ke, Ping Hu, Chenghao Yang, Renbao Zhang

**Affiliations:** School of Mechanical and Electrical Engineering and Automation, Shanghai University, Shanghai 200444, China; xxke@staff.shu.edu.cn (X.K.); jingzhanhui2017@163.com (C.Y.); Zz3476502133@163.com (R.Z.)

**Keywords:** human–machine interaction, Seq2Seq, NLP, deep learning, context semantic coding

## Abstract

During multi-turn dialogue, with the increase in dialogue turns, the difficulty of intention recognition and the generation of the following sentence reply become more and more difficult. This paper mainly optimizes the context information extraction ability of the Seq2Seq Encoder in multi-turn dialogue modeling. We fuse the historical dialogue information and the current input statement information in the encoder to capture the context dialogue information better. Therefore, we propose a BERT-based fusion encoder ProBERT-To-GUR (PBTG) and an enhanced ELMO model 3-ELMO-Attention-GRU (3EAG). The two models mainly enhance the contextual information extraction capability of multi-turn dialogue. To verify the effectiveness of the two proposed models, we demonstrate the effectiveness of our model by combining data based on the LCCC-large multi-turn dialogue dataset and the Naturalconv multi-turn dataset. The experimental comparison results show that, in the multi-turn dialogue experiments of the open domain and fixed topic, the two Seq2Seq coding models proposed are significantly improved compared with the current state-of-the-art models. For specified topic multi-turn dialogue, the 3EAG model has the average BLEU value reaches the optimal 32.4, which achieves the best language generation effect, and the BLEU value in the actual dialogue verification experiment also surpasses 31.8. for open-domain multi-turn dialogue. The average BLEU value of the PBTG model reaches 31.8, the optimal 31.8 achieves the best language generation effect, and the BLEU value in the actual dialogue verification experiment surpasses 31.2. So, the 3EAG model is more suitable for fixed-topic multi-turn dialogues for the two tasks. The PBTG model is more muscular in open-domain multi-turn dialogue tasks; therefore, our model is significant for promoting multi-turn dialogue research.

## 1. Introduction

Language communication is an integral part of people’s daily life. With the development of artificial intelligence technology and natural language processing, the research on the human–machine dialogue has been transformed from single question–answer dialogue to multi-turn dialogue, which is more challenging. The applied dialogue model is concerned; it divides into two types in broadly human–machine dialogue [1]. The first is a task-based dialogue, and the second is an open-domain dialogue. Task-oriented dialogue is mainly task driven, and the machine needs to understand, ask, clarify to deal with users’ needs. Task-based dialogue topics are relatively fixed and generally have poor generalization ability, but they have more advantages than non-task-based dialogues when dealing with questions–answers tasks. For non-task-based dialogues, they break through the topic limitation [2]. They can provide better responses between multiple topics and even in open domains, making human–machine dialogue resemble the natural communication between people. Still, the resulting methodological research is also more challenging. The research methods of non-task-based dialogue models divide into retrieval-based methods and neural generation-based methods. The retrieval-based method mainly completes the reply matching of each dialogue turn, a discriminative model. The neural generation-based models mainly include Sequence-to-Sequence (Seq2Seq) Models [3,4,5], Dialogue Context [6], Response Diversity [7,8], Topic and Personality [9,10], Outside Knowledge Base. Dialogue context, Response Diversity, Topic or Personality, and other methods adopt multi-classification of contextual dialogues and then select and integrate the best alternative answers to return. This method is, in principle, a classification language model. At the same time, Outside Knowledge Base needs to build a massive amount of knowledge library to adapt to the diversity of the dialogue process. Sequence-to-Sequence models reduce manual preprocessing and post-processing. It tries to make the model from the original input to the final output as effective possible, giving the model more space for automatic adjustment according to the data. It has been widely used in various dialogue generation research [11,12] due to its advantages, such as increasing the model’s overall fit [10].

Although many scholars have made some improvements and optimizations, the Transformer model [12] proposed by Google researchers has promoted the development of the Natural Language Generation. It offered a vast improvement; subsequently, the BERT model proposed by Devlin et al. [13] has become a natural language encoder in Seq2Seq. Later, with the proposal of the GPT model [14], the attention mechanism is used in the field of natural language generation. It has achieved excellent performance and significantly promoted the progress of the generation task. To combine the advantages of BERT and GPT, the MASS model proposed by Song [15] of Microsoft Research Asia shields some of the sentences and then regenerates this fragment in the decoder. They can easily change the structure of the model by adjusting the hyperparameters, but their specific achievements in multi-turn dialogue are still unknown. Still, the innovation of the model has expanded. The optimization of these related models is undoubtedly the transformation of the encoder and decoder. In any case, they still cannot rival the position of Seq2Seq in the field of Natural Language Generation.

The communication mode of multi-turn dialogue is widespread in daily communication. Table 1 shows an example of multi-turn dialogue in Tencent AI Lab [16]; the research of multi-turn dialogue has experienced template matching, task-driven dialogue, recommendation model dialogue management, Knowledge Graph, Seq2Seq, and other research processes. Template matching [17,18] completes the dialogue communication by building a vast multi-turn dialogue database to retrieve information. The task-driven dialogue [19,20,21] is mainly in the task-oriented dialogue field, such as booking an airline ticket and a hotel, and its context fills with fixed sentences. For the recommendation model dialogue management [22], it uses information, such as the extraction of input features, and historical conversations uses a search or retrieval model to extract the optimal answer. For knowledge graph-based dialogue, which manages the conversation by constructing a knowledge graph, Xu [23] disassembled the multi-turn open-domain dialogue into two sub-tasks: planning the dialogue target sequence and the in-depth dialogue for a given dialogue target for the first time. The knowledge graph-based dialogue introduces displayed and interpretable dialogue states and actions for dialogue policy learning, which facilitates the design of Reward factors related to dialogue goals and uses dialogue goals and fine-grained topics to guide response generation. Finally, the multi-turn generation of Seq2Seq [24,25] mainly takes the current sentence input plus historical context information and sends it to the encoder, then decodes it through the decoder to generate a reply. Wu [26] et al. developed sentence information, which is encoded using tokens in the encoder encoding process and then combined with the token information in the decoding process to generate dialogue.

## 2. Related Work

Google’s Oriol Vinyals [27] first proposed a neural network dialogue model, which is the source of Seq2Seq. Then, Li Hang et al. [28] first applied the Seq2Seq translation model with attention to dialogue tasks based on Weibo comment data. Baidu and Université de Montréal [29,30] successively adopted the Seq2Seq framework to generate the Nth sentence using the first N-1 sentences, which divided the dialogue model into two layers. The entire dialogue in the first layer combines all penalties and the second layer. Each dialogue is a combination of all words. Still, the author believes that the fundamental reason for the low quality of dialogues generated by language models, such as RNNLM, is that the model does not deal with the random features and noise hidden in the dialogue. Hence, the following sentence causes the dialogue. The effect is not ideal, so the context layer RNN and the hidden state layer are embedded in the middle of Seq2Seq to improve the overall dialogue randomness. Université de Montréal, Georgia Institute of Technology, Facebook, and Microsoft Research [31] jointly trained a data-driven open-domain dialogue model. They believed that the current user query sentence and historical dialogue information should be considered when generating the current dialogue. This dialogue generation model has been generalized in open-domain multi-turn dialogue research. To solve the generation of meaningless sentences in Seq2Seq dialogue generation, Li Ji et al. [32] proposed to train Seq2Seq with maximum mutual information, which effectively solved the problem of generating irrelevant replies. However, it uses a traditional network model, which is more sensitive to the sequence length. This paper mainly combines the current popular pre-training mechanism to improve the semantic fusion effect in the multi-turn dialogue generation process.

## 3. Coding Model for Multi-Turn Dialogue

Since our work involves the problem of word vector representation, we tried Word2Vec [33] word vector training method and GolVe [34] word vector training method for word vector representation. These two types of model methods for word vector representation adopt fixed expressions. They are trained based on single corpus sentences. The word vector information of each word only captures all the information of the sentence, and the context information is relatively lacking. In addition, the ELMO [35] pre-training word vector method captures the word vector information of the dialogue model relatively well and can grasp the sentence semantics of the context better, so we used ELMO to represent the word vector.

The early structure of the traditional Seq2Seq dialogue model mentioned in this paper combines bidirectional GRU [36] and unidirectional GRU models. After the Transformer model was proposed, the dialogue model’s coding part adopts the attention mechanism to capture the context information. Compared with the previous encoder, the capture ability has dramatically improved. After the BERT masked language model proposal, it adopts the context prediction method. The method has continuously enhanced the ability to generate word vectors and capture information, such as syntax and semantics.

This research focused on the context management model and word vector encoding learning optimization performance in generative dialogue. The improved encoding model based on BERT applied multi-turn dialogue. For the first time, we propose to embed the multi-turn contextual positional encoding into the BERT model, which helps to improve the generator’s decoding performance.

Our models were divided into two forms: (1) multi-turn word-sentence vector encoding model, contextual syntactic and semantic capture for historical multi-turn dialogues; (2) PBTG network model, for historical conversation semantic multi-sentence information encoding the device model, jointly encodes the historical sentence information and current input sentence.

### 3.1. Context Semantic Encoding Model

#### 3.1.1. PBTG Context Semantic Coding Model

**Figure 1:**
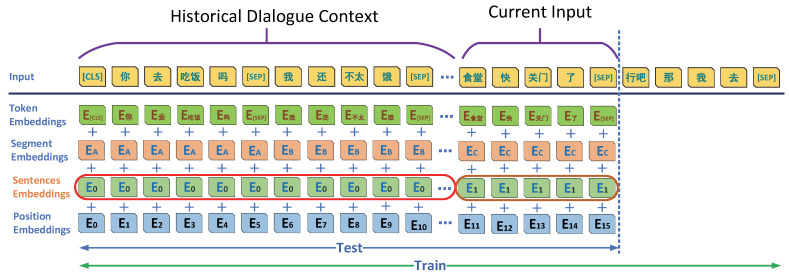
Context Encoding Structure of the PBTG Model.

We used historical turn-based sentence coding combined with the current input sentence to encode multi-turn contextual word coding. Based on the word encoding of BERT [13], the historical turn sentence information and the current input information encoding were added. It started from the first turn of dialogue: the first sentence started with [“CSL”] and ended with [“SEP”] between each turn, where the encoding result was the vector sum of Token encoding, Segment encoding, Sentences encoding, and Position encoding. Furthermore, the model’s architecture is shown in Figure 1.

Among the parameters, “Historical Dialogue” is all the historical information of the dialogue from the first round; “Current Input” is the input dialogue sentence of the current turn, followed by the decoder input information of teacher forcing; the encoding result is presented in Equation (1).

(1) $$W_{E}=f_{Token}(input)⊕f_{Seg}(input)⊕f_{Sent}(input)⊕f_{Posi}(input)$$

#### 3.1.2. Context Semantic Encoding Structure of 3EAG Model

**Figure 2:**
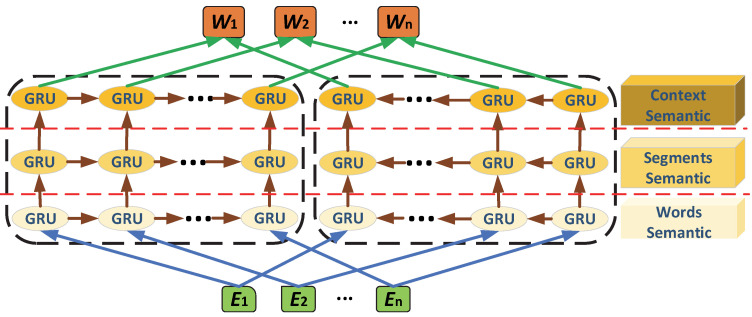
Context Semantic Encoding Structure of the 3EAG Model.

The traditional ELMO [35] model pre-training adopts a bidirectional two-layer LSTM [37] model to capture contextual information. The contextual information mainly includes the context in a single sentence and lacks information capture between sentences. Therefore, this study used the traditional ELMO model to capture contextual information. The number of layers was increased to capture context information, and a 3-layer GRU [36] bidirectional network as used to capture word information, segment information, and context information. The model’s structure is shown in Figure 2.

For the context semantic encoding of 3EAG, its forward model was:

(2) $$p(w_{1},w_{2},....,w_{N})=\overset{N}{\underset{k=1}{\prod }}p(w_{k}|w_{1},w_{2},....,w_{k-1}).$$

The backward model was:

(3) $$p(w_{1},w_{2},....,w_{N})=\overset{N}{\underset{k=1}{\prod }}p(w_{k}|w_{k+1},w_{k+2},....,w_{N}).$$

The context semantic encoding output $W_{ELMo,k}$ was:

(4) $$W_{ELMo,k}=W_{words,k}⊕W_{segs,k}⊕W_{context,k}$$

(5) $$\left\{ \begin{array}{l} W_{words,k}=\{x_{k}^{LM},{\vec{h}}_{k,j}^{LM},{\overset{\leftarrow }{h}}_{k,j}^{LM}|j=1\}\\ W_{Segs,k}=\{x_{k}^{LM},{\vec{h}}_{k,j}^{LM},{\overset{\leftarrow }{h}}_{k,j}^{LM}|j=2\}\\ W_{context,k}=\{x_{k}^{LM},{\vec{h}}_{k,j}^{LM},{\overset{\leftarrow }{h}}_{k,j}^{LM}|j=3\} \end{array}\right.$$

where k is the position of each word in the coder, with a value range in {0,1,2,..,N}.

The encoding model adopted a three-layer Markov chain model, and its semantic representation depended on the dialogue information of the previous turn. Due to the hierarchical dialogue structure, the contextual correlation is not high in multi-turn dialogues with uncertain topics [38,39]. We tried to conduct experiments in open-domain and fixed-topic multi-turn dialogues. The follow-up experimental results also prove that it is more suitable for fixed-topic multi-turn dialogues. This paper performed a comparative investigation in the fixed-topic multi-turn dialogue experiment and the open-domain dialogue experiment. The detailed results are shown in the experimental Results Analysis Section.

### 3.2. Encoder Network Model

#### 3.2.1. PBTG Network Model

**Figure 3:**
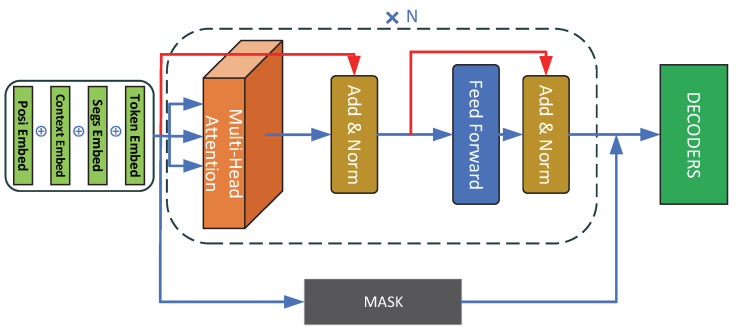
PBTG model structure.

In this study, the contextual sentence encoding result was used as the input of the BERT model, and the Encoder part of the Transformer [6] model as the model framework. The model structure is shown in Figure 3. This paper tested the coding effect of the number of model layers in a variety situations.

#### 3.2.2. EAG Network Model

**Figure 4:**
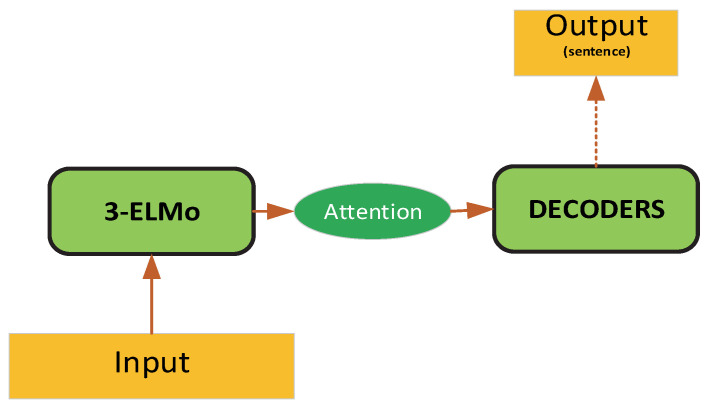
3EAG model structure.

We also used the ELMO variant model as the research effect of the encoder. First, the 3-layer forward and backward bidirectional GRU [36] model captured the context information. The Attention mechanism and input jointly encoded the historical information and the current input as DECODERS. The model structure is shown in Figure 4.

## 4. Experiments

This section describes the experiments conducted on multi-turn dialogue data and shows the promising results.

### 4.1. The Data Set

**Figure 5:**
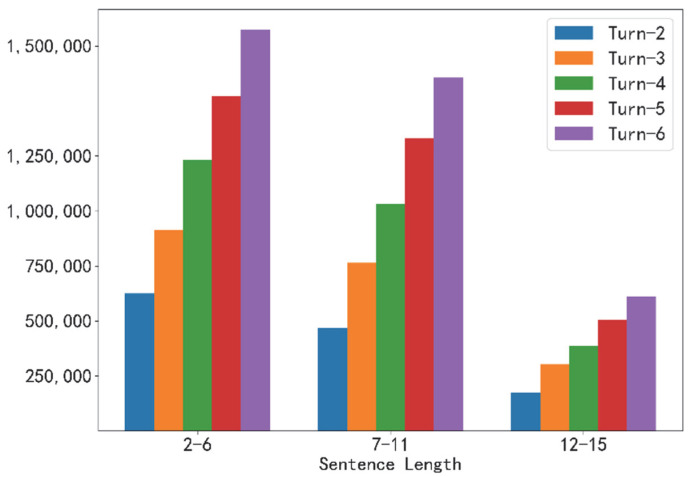
Sentence length distribution of the data sets.

Our experimental data adopts the open-source LCCC-large [40] Chinese multi-turn dialogue dataset of Tsinghua University and the open-source Naturalconv [16] multi-turn dialogue dataset of Tencent AI Lab. We mainly cleaned the two data comprehensively, and the total number of Naturalconv databases is 19,919. When we split multiple turns, we expanded the data into 2\3\4\5\6 by splitting and combining each turn with a data volume of 50,000. Our final data contained 2~6 turns of dialogue data, and the processed data had a data volume of 170,000 per turn; the total population was 850,000 (counting the dialogue example in Table 1 as a data volume of 6 turns). The number of sentences after processing is shown in Table 2. The overall sentence length distribution is shown in Figure 5.

For the fixed topic dialogue experiment, we screened out 50,000 dialogues on sports topics, health topics, and science and technology topics for experiments. Two thousand pieces of dialogue data were used as verification dialogues. The statistics of our processed sentences are shown in Table 3.

### 4.2. Experimental Parameters and Results Analysis

#### 4.2.1. Experimental Parameters

For the experiments with the 3EAG model, we adopted the context-encoded representation $E\in R^{M\times N}$ as the model input representation. The single input of sentence length was increased to 15, and the word vector dimension was set to 512. We used the forward and backward three-layer GRU combined with self-attention as an encoder, while the decoder used a three-layer unidirectional GRU.

For the experiments with the PBTG model, we adopted the context-encoded representation $E\in R^{M\times N}$ as the model input representation. The single input sentence length was increased to 15, and we set the word vector dimension to 512. We set the number of ENCODER layers to 8–12 layers, the number of attention heads to 8, the masking rate of MASK to 0.17, and the DECODER layer to use four layers of unidirectional GRU as the generator.

Based on the above parameter settings, we testes the language effects of the traditional Seq2Seq (LSTM to LSTM) model, Transformer model, and our 3EAG model and PBTG model in open-domain dialogue generation and fixed-topic dialogue generation.

In this experiment, BLEU [41] was used to judge the quality of the model generation effect. BLEU is one of the commonly used evaluation indicators of the Seq2Seq model. With an improvement in the effect, we used the BLEU value to evaluate the similarity between the response generated by the model and the target sentence. We used BLEU-2, BLEU-3, and BLEU-4 for the specific evaluation in this experiment.

#### 4.2.2. Results Analysis

As shown from Table 4, the average BLEU value of our 3EAG model is 2.3 higher than the traditional Seq2Seq and 0.4 higher than the average BLEU value of the Transformer model. The average BLEU value of our improved PBTG model is 3.3, 1.4, and 1.0 higher, respectively, compared with the conventional Seq2Seq, Transformer, 3EAG the models. In the actual dialogue verification experiments, our average BLEU value also achieves a score of 31.2, which outperforms the previous three models. It can be seen from these results that our model has a stronger ability to identify topics in the contextual information capture ability in multi-turn dialogue generation.

For the PBTG model, we tested the encoding performance between encoder layers 8–12, as shown in Table 5.

**Figure 6:**
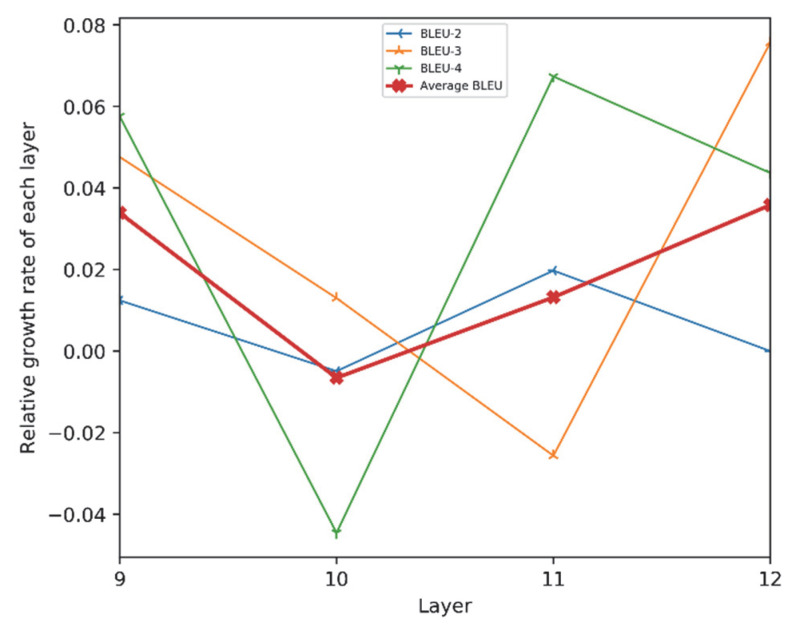
The growth rate of BLEU value varies with the number of network layers.

Table 5 and Figure 6 show that, in open-domain dialogue generation, for our PBTG model, the number of layers of the encoder network is between 8–12 layers, and its BLEU value keeps increasing as the number of layers increases. Additionally, the changes of BLEU-2, BLEU-3, and BLEU-4 all show an upward trend; their growth rates are also stable in the region 0~0.08. It can be seen that the more layers the encoder has, the better the generation effect of the model. From the experimental results, we can see the effectiveness of our two encoding models for the multi-turn dialogue generation task.

For multi-turn dialogue experiments with fixed topics, we used the same experimental parameters to conduct experiments, and the obtained BLEU performance is shown in Table 6.

From the multi-turn dialogue experiments on fixed topics, we can conclude that the PBTG model and 3EAG model proposed in this paper achieved better results. Their average BLEU values are higher than the previous two models; PBTG model is better than Seq2Seq and Transformer, respectively. The model outperformed the other two models by 0.9 and 0.5, respectively, while the average BLEU improvement of 3EAG was 3.1 and 1.7, respectively. Finally, we conducted an actual dialogue verification experiment on the 3EAG model. According to the experimental results, the BLEU-4 value of the actual dialogue reached 21.0, and the average BLEU value also achieved a good score of 31.8.

Through the above open-domain and fixed-topic multi-turn dialogue experiments, we can conclude that, for the two models proposed in this paper, PBTG and 3EAG, the BLEU-2, BLEU-3, and BLEU-4 values of PBTG were the best experimental results (in the open domain dialogue experiment), and the average BLUE value was 1.0 higher than that of 3EAG. Therefore, the PBTG model is more suitable for open-domain dialogue tasks and has the best effect. In the fixed-topic dialogue experiment, the BLEU-2, BLEU-3, and BLEU-4 values of 3EAG obtained the best experimental results, and the average BLUE value was 0.8 higher than that of 3EAG. The average BLEU value of the actual dialogue experiment also achieved better results. The value of 31.8 is excellent, so the 3EAG model is more suitable for fixed-topic dialogue tasks and has the best effect.

## 5. Conclusions

Aiming to achieve a multi-turn dialogue generation system model, we proposed a semantic encoding model as the inner encoder of the dialogue generation model. It improves the ability of contextual semantic extraction and can integrate historical dialogue information and current input information in multi-turn dialogue, which enhances the dialogue context. We combined the LCCC-large multi-turn dialogue dataset and Naturalconv multi-turn data for our research purpose. We, then, adopted the combined split method to construct our open-domain and fixed-topic multi-turn datasets. Additionally, to extract contextual semantics in multi-turn dialogues, we proposed two contextual semantic fusion models, 3EAG and PBTG. To further validate the performance of our model, we evaluated it on our multi-turn dialogue dataset. The experimental results show that our proposed 3EAG model achieves the optimal language effect for fixed-topic dialogues. The PBTG model achieves the best dialogue effect in the open-domain dialogue generation experiment and verifies the effectiveness of dialogue context information extraction and related model design. Therefore, the model we proposed has a good significance for promoting multi-turn dialogue research. We will carry out in-depth research in the direction of multi-turn emotional dialogue and topics recognition of multi-turn dialogue in the future to test the capabilities of our model.

## Figures and Tables

**Table 1:** Examples of multi-turn dialogue.

Turns	Dialogue Text
Turn-1	hi~你好啊 (Hello)
Turn-2	嗯，你好，有什么事吗? (Hello, do you have any questions?)
Turn-3	看你一个人也挺无聊的，来聊会天吧。(You look bored, let us have a chat.)
Turn-4	好啊，聊点什么呢？(OK, what shall we talk about?)
Turn-5	你看网球吗，我可是很喜欢网球的。(Do you like tennis? I like it very much.)
Turn-6	网球啊，一般吧，我就知道个李娜。(Tennis is OK. I only know Li Na.)

**Table 2:** Data sources and processing results.

Data Sources	Turns	Data Quantity
Naturalconv [10]	2; 3; 4; 5; 6	50,000; 50,000; 50,000; 50,000; 50,000
LCCC-large [26]	2; 3; 4; 5; 6	120,000; 120,000; 120,000; 120,000; 120,000
**Ours**	**2; 3; 4; 5; 6**	**170,000; 170,000; 170,000; 170,000; 170,000**

**Table 3:** Statistics of conversation data on fixed topics.

Topics	Turn	Data Quantity
Sport	2; 3; 4; 5; 6	10,000; 10,000; 10,000; 10,000; 10,000
Health	2; 3; 4; 5; 6	10,000; 10,000; 10,000; 10,000; 10,000
Tech	2; 3; 4; 5; 6	10,000; 10,000; 10,000; 10,000; 10,000
Verification	random	2000

**Table 4:** BLUE’s evaluation results of open-domain dialogue.

Model	BLEU-2	BLEU-3	BLEU-4	Average BLEU
Seq2Seq	39.2	29.1	17.3	28.5
Transformer	40.3	31.4	19.5	30.4
**3EAG(Our)**	**40.7**	**31.6**	**20.2**	**30.8**
**PBTG(Our)**	**41.3**	**32.7**	**21.5**	**31.8**
**PBTG** **(Verification)**	**40.9**	**32.1**	**20.7**	**31.2**

**Table 5:** BLEU performance of different layers for PBTG’s encoders.

#Layers	BLEU-2	BLEU-3	BLEU-4	Average BLEU
8	40.2	29.4	19.1	29.5
9	40.7	30.8	20.2	30.5
10	40.5	31.2	19.3	30.3
11	41.3	30.4	20.6	30.7
**12**	**41.3**	**32.7**	**21.5**	**31.8**

**Table 6:** BLUE’s evaluation results of fixed topics.

Model	BLEU-2	BLEU-3	BLEU-4	Average BLEU
Seq2Seq	40.4	29.5	18.2	29.3
Transformer	41.2	31.7	19.3	30.7
PBTG(Our)	41.4	31.9	20.3	31.2
**3EAG(Our)**	**42.6**	**33.2**	**21.6**	**32.4**
**3EAG** **(Verification)**	**42.1**	**32.5**	**21.0**	**31.8**
